# Prevalence and underreporting of immunization errors in childhood vaccination: results of a household survey

**DOI:** 10.1590/1980-220X-REEUSP-2023-0253en

**Published:** 2024-02-19

**Authors:** Stênio Henrique Oliveira, Brener Santos Silva, Lívia Maria Rezende Carvalho, Tarcísio Laerte Gontijo, Ione Carvalho Pinto, Eliete Albano de Azevedo Guimarães, Valéria Conceição de Oliveira

**Affiliations:** 1 Universidade Federal de São João del-Rei, Programa de Pós-Graduação em Enfermagem, Divinópolis, MG, Brazil.; 2 Universidade do Estado de Minas Gerais, Departamento de Ciências da Reabilitação e Saúde, Divinópolis, MG, Brazil.; 3 Universidade de São Paulo, Escola de Enfermagem de Ribeirão Preto, Programa de Pós-Graduação Enfermagem em Saúde Pública, Ribeirão Preto, SP, Brazil.

**Keywords:** Drug-Related Side Effects and Adverse Reactions, Nursing, Medication Errors, Immunization, Patient Safety, Vaccination, Efectos Colaterales y Reacciones Adversas Relacionados con Medicamentos, Enfermería, Errores de Medicación, Inmunización, Seguridad del Paciente, Vacunación, Efeitos Colaterais e Reações Adversas Relacionados a Medicamentos, Enfermagem, Erros de Medicação, Imunização, Segurança do Paciente, Vacinação

## Abstract

**Objective:**

To investigate underreporting of immunization errors based on vaccination records from children under five years of age.

**Method:**

An epidemiological, cross-sectional analytical study, carried out through a household survey with 453 children aged 6 months to 4 years in three municipalities in Minas Gerais in 2021. A descriptive analysis was carried out, and the prevalence of the error was calculated per 100 thousand doses applied between 2016 and 2021. The magnitude was estimated of the association between variables by prevalence and 95% Confidence Intervals (95%CI). To analyze underreporting, State reporting records were used.

**Results:**

A prevalence of immunization errors was found to be 41.9/100,000 doses applied (95%CI:32.2 – 51.6). The highest prevalence occurred between 2020 (50.0/100,000 doses applied) and 2021 (78.6/100,000 doses applied). The most frequent error was an inadequate interval between vaccines (47.2%) associated with adsorbed diphtheria, tetanus and pertussis (DTP) vaccine (13.7/100,000) administration. Vaccination delay was related to immunization errors (7.55 95% CI:2.30 – 24.80), and the errors found were underreported.

**Conclusion:**

The high prevalence of underreported errors points to a worrying scenario, highlighting the importance of preventive measures.

## RESUMO

**Objetivo:**

Investigar a subnotificação de erros de imunização a partir dos registros de vacinação da caderneta de crianças menores de cinco anos.

**Método:**

Estudo epidemiológico, transversal analítico, realizado por inquérito domiciliar com 453 crianças de 6 meses a 4 anos em três municípios de Minas Gerais em 2021. Realizaram-se a análise descritiva e o cálculo da prevalência do erro por 100 mil doses aplicadas entre 2016 e 2021. Estimou-se a magnitude da associação entre as variáveis pela prevalência e Intervalos de Confiança 95% (IC95%). Para a análise da subnotificação, utilizaram-se os registros de notificação do Estado.

**Resultados:**

Encontrou-se uma prevalência de erros de imunização de 41,9/100.000 doses aplicadas (IC95%:32,2 – 51,6). A maior prevalência ocorreu entre 2020 (50,0/100.000 doses aplicadas) e 2021 (78,6/100.000 doses aplicadas). O erro mais frequente foi intervalo inadequado entre vacinas (47,2%) associado à administração da vacina adsorvida difteria, tétano e pertussis (DTP) (13,7/100.000). O atraso vacinal relacionou-se ao erro de imunização (7,55 IC95%:2,30 – 24,80), e os erros encontrados foram subnotificados.

**Conclusão:**

A alta prevalência de erros subnotificados aponta para um cenário preocupante, ressaltando a importância de medidas preventivas.

## RESUMEN

**Objetivo:**

Investigar el subregistro de errores de vacunación a partir de los registros de vacunación de niños menores de cinco años.

**Método:**

Estudio epidemiológico, analítico transversal, realizado mediante encuesta de hogares con 453 niños de 6 meses a 4 años en tres municipios de Minas Gerais en 2021. Análisis descriptivo y cálculo de la prevalencia de error por 100 mil dosis aplicadas entre 2016 y 2021. La magnitud de la asociación entre las variables se estimó mediante prevalencia e intervalos de confianza del 95% (IC95%). Para analizar el subregistro se utilizaron los registros de notificaciones estatales.

**Resultados:**

Se encontró una prevalencia de errores de inmunización de 41,9/100.000 dosis aplicadas (IC95%: 32,2 – 51,6). La prevalencia más alta se produjo entre 2020 (50,0/100.000 dosis aplicadas) y 2021 (78,6/100.000 dosis aplicadas). El error más frecuente fue un intervalo inadecuado entre vacunas (47,2%) asociado a la administración de la vacuna adsorbida contra la difteria, el tétanos y la tos ferina (DTP) (13,7/100.000). El retraso en la vacunación estuvo relacionado con errores de vacunación (7,55 IC 95%: 2,30 – 24,80), y los errores encontrados fueron subreportados.

**Conclusión:**

La alta prevalencia de errores no reportados apunta a un escenario preocupante, destacando la importancia de las medidas preventivas.

## INTRODUCTION

Although the benefits of vaccination are numerous, the complexity and constant changes in vaccination schedule, the inclusion of new immunobiological agents^(1–3)^, the similarity of vials and presentation of vaccines^(4,5)^, in addition to structural and organizational obstacles and management in vaccination rooms^(4)^, predispose to the risk of immunization errors.

An immunization error can be defined as any preventable event resulting from errors in immunobiological agent preparation, handling or administration, which can cause harm to patients, reducing or nullifying the expected effect of vaccination^(6,7)^.

An immunization error can trigger an Event Supposedly Attributable to Vaccination or Immunization (ESAVI), which may be capable of generating a negative impact on public health, increasing costs for health services, and reducing the population’s confidence in immunization programs^(1,8)^ and increase vaccine hesitancy^(8)^, directly affecting vaccination coverage and the control and eradication of vaccine-preventable diseases.

Even though it is an extremely relevant subject, studies point to an underreporting of immunization errors that has not yet been measured in health services^(9–11)^. This condition can compromise the adoption of preventive measures by managers who, due to lack of knowledge of the real extent of the problem, assume that errors are not occurring, directly contributing to their occurrence and maintenance(1).

Most of studies already carried out in Brazil, on the prevalence/incidence of immunization errors, used reports registered in the secondary database of the Adverse Events Following Immunization Information System (SIEAPV – *Sistema de Informação de Eventos Adversos Pós*-*Vacinação*) of the Brazilian National Immunization Program (PNI – *Programa Nacional de Imunizações*)^(2,9,10,12,13)^ and, for this reason, may not demonstrate the reality faced in health services. These reports point to lack of quality of information recorded in investigation forms, typing errors, incomplete fields and underreporting^(9,10,12,13)^.

The importance of reporting immunization errors by health professionals, with complete data, is highlighted, which must be perceived as essential for developing control strategies in order to avoid new occurrences, directly influencing quality of care(14).

It is believed that there is underreporting of immunization errors, making it difficult to visualize the real proportion of immunization errors, which is higher than that presented in secondary studies. Considering the above, the study aimed to investigate the underreporting of immunization errors based on vaccination records from children under five years of age.

Carrying out this study advances knowledge by proposing a household survey to directly search for data, enabling knowledge of underreported immunization errors that affect children, envisioning the adoption of preventive measures that minimize the possibility of the same happening. Furthermore, given the current scenario of dissemination of fake news and increased vaccine hesitancy^(8,15)^, factors that weaken PNI, such as immunization errors, must be investigated.

## METHOD

### Study Design

This is an analytical, cross-sectional observational study, based on the STrengthening the Reporting of OBservational studies in Epidemiology guidelines, carried out through a home-based vaccination survey.

### Study Site

The study was carried out in three municipalities in the Western macro-region of the state of Minas Gerais, Brazil. The West macro-region of Minas Gerais is one of the 14 macro-regions of the state, formed by the union of 53 municipalities^(16)^. It has a vast territorial extension, with 31,543 km^2^, a medium-high Human Development Index, with a diversified economy(17).

To select the municipalities, reports of immunization errors from municipalities in the macro-region in 2019 were considered. According to data provided by the State Department of Health of Minas Gerais, 34 silent municipalities were identified (which did not report an immunization error in 2019). To compose the municipalities participating in the study, one municipality of each population size identified as A, B and C was drawn.

### Population, Selection Criteria and Sample Definition

The study population consisted of children aged between 6 months and 4 years 11 months and 29 days (4y 11m 29d). Children residing in the municipality and having vaccination records at the time of data collection were included. Non-resident children who were at home at the time of data collection, even if they were of eligible age, residents of the visited household who did not present proof of vaccination and/or were not accompanied by a legal guardian (18 years of age or older or emancipated by law), were excluded.

To calculate sample size in each municipality, the total population of children aged 0 to 4 years old was considered, according to data from the Live Birth Information System, and a prevalence of immunization error of 10.96 in the West macro-region in 2019 for 100,000 doses applied^(18)^. Considering a 90% agreement proportion and a 95% confidence level, the number of children to be interviewed was 135 in municipality A, 112 in municipality B and 66 in municipality C, adding approximately 10% to avoid losses, reaching a final sample of 435 children.

Sampling plan was conducted through two-stage sampling, considering the number of Basic Health Units (BHU) and the number of children per coverage area. The first stage unit consisted of all BHU in each municipality. In the second selection stage, children between 6 months and 4 years old were drawn with a probability proportional to their size, measured by the number of children registered in each BHU.

### Data Collection

Data collection was carried out between June and October 2021, through on-site interviews by researchers graduated in health who used tablets with a structured form inserted into *Google Forms^®^*. Collection included the participation of Community Health Workers (CHW), helping to locate children and introducing the team to families. Families were approached at least twice, before being considered a loss (due to impossibility of contact) or a refusal.

A structured data questionnaire was used containing children’s identification, age, sex and skin color self-reported by parents and personal data such as children’s Individual Registry and Brazilian Health System Card (SUS Card). Moreover, a photocopy of children’s vaccination record pages was collected for further analysis of immunization error.

### Data Analysis and Treatment

The identification of immunization errors and their reporting occurred in five stages, after the end of data collection in each municipality: 1) booklet photocopy analysis by two researchers in a double-blind manner to identify the presence of an immunization error; 2) review of data regarding errors found by both researchers; 3) assessment of errors found by a vaccination expert researcher; 4) checking the date of administration of the dose affected by an immunization error in the Citizen’s Electronic Record (PEC e-SUS), in order to ensure that there was no recording error in children’s record; 5) checking, through the technical reference on immunization of the Minas Gerais State Department of Health, reports of immunization errors in the PNI Information System in SIEAPV and e-SUS *Notific*a module.

In addition to analyzing the immunization error, the presence of a vaccination delay was verified for any vaccine registered on the cards. A dose received more than 30 days after scheduling date was considered delayed^(19)^. This study hypothesizes that vaccine delay and children’s age are factors associated with immunization errors.

The errors that could be identified on the cards, as recommended by the Epidemiological Surveillance Manual for Adverse Events Following Immunization, were inadequate interval between doses, inadequate interval between vaccines and vaccine administered outside the recommended age^(6,20)^. Immunization errors were not assessed in vaccines administered in special situations, campaigns and zero dose of (attenuated) measles, mumps and rubella (MMR) vaccine.

For analysis, the recommended vaccination schedules and intervals described in the Normative Instruction referring to the Brazilian National Vaccination Calendar^(20)^ were considered, in addition to the changes made to the calendar between 2016 and 2021, highlighting the expansion of (inactivated) adsorbed hepatitis A vaccine (HepA) for children under 5 years of age in 2017, the inclusion of the 2^nd^ dose of chickenpox vaccine (CV) in 2018 and the introduction of the 2^nd^ dose of (attenuated) yellow fever vaccine (YFV) in 2020.

Furthermore, in order not to overestimate the results of the analysis, immunization errors were disregarded for doses administered four days before the recommended interval or minimum age, according to Brazilian PNI criteria^(21)^, and doses of YFV, SCR and CV, administered at an interval between 15 and 29 days between them(20).

To analyze the reporting of immunization errors found in vaccination records, two sources of information were used: PNI Information System in the SIEAPV module and e-SUS *Notifica*, as, from January 2021, reports began to be carried out, obligatorily, in this information system. Consultations to these information systems were carried out by technical reference in immunization of the State Department of Health of Minas Gerais.

A file was generated in Microsoft Excel^®^ with all information regarding immunization errors (children’s name, age, date of birth, SUS Card, CPF, mothers’ name, municipality, vaccines related to immunization error with respective dates of administration and classification of immunization error) to identify the reporting of these errors in information systems.

A descriptive data analysis was performed, including absolute (n) and relative frequencies (%). To calculate the prevalence of immunization errors per 100,000 doses administered, the total number of errors found in vaccination records (numerator) and the number of doses administered during the study period (denominator) were considered, according to the birth date of older children and the end of data collection. The doses administered during the period were extracted from the Brazilian PNI Information System.

Bivariate binary logistic regression analysis was also carried out using the IBM^®^ SPSS^®^ Statistics 10 software, considering immunization error and covariates as the outcome variable, for calculating Pearson’s chi-square test and Confidence Interval. The magnitude of the association between the presence of immunization error and covariates “vaccination delay”, “skin color”, “child age” and “sex” was estimated using the Prevalence Ratio (PR) and 95% Confidence Intervals (_95%_CI).

### Ethical Aspects

This study is part of a larger project entitled “*Avaliação dos erros de imunização e proposta de intervenção*”, financed in Call MCTIC/CNPq 28/2018 – Universal. It was approved by the Research Ethics Committee of the *Universidade Federal de São João del-Rei*, Center-West *Campus*, under Opinion 3.817.007 and with amendment approved under 4,657,136, in addition to Certificate of Presentation for Ethical Consideration (CAAE – *Certificado de Apresentação para Apreciação Ética*) 23888819.9.0000.5545.

## RESULTS

A total of 607 homes were visited, 102 of which were closed. Five parents/guardians refused to participate and 47 children were excluded because they did not have the child’s booklet containing vaccination records at the time of data collection. Thus, 453 children participated in the study, divided into 228 children in municipality A, 149 children in municipality B and 76 in municipality C. Regarding the characteristics of the 453 children assessed, it was observed that 230 (50.8%) were female, were 1 year old (28.0%) and almost half of them (223; 49.2%) had their color/race self-reported by their parents as brown.

Furthermore, 55 immunization errors were found in the 453 vaccination records assessed. The number of children affected by these errors was 44 (9.7%), with 36 (81.8%) affected by one immunization error, 7 (15.9%) by two immunization errors and 1 (2.3%) child was affected by 5 immunization errors, data not presented in table.

Inadequate interval between vaccines (47.3%) and vaccine administered outside the recommended age (41.8%) were the most frequent errors. Regarding vaccination delays, 307 (67.8%) cards showed some vaccine administered late. Of the vaccination records of the 44 children interviewed who suffered an immunization error, in 41 (93.2%) records there was a delay in vaccination (Table 1).

**Table 1:** Descriptive analysis of vaccination records assessed, Minas Gerais, Brazil, 2021.

Variables	Municipality A n (%)	Municipality B n (%)	Municipality C n (%)	Total n (%)
**Sex (n = 453)**				
Male	125 (54.8)	69 (46.3)	36 (47.4)	230 (50.8)
Female	103 (45.2)	80 (53.7)	40 (52.6)	223 (49.2)
**Child’s age (n = 453)**				
From 6 months to < 1 year	39 (17.1)	24 (16.1)	6 (7.9)	69 (15.3)
1 year	62 (27.2)	40 (26.8)	25 (32.9)	127 (28.0)
2 years	48 (21.1)	32 (21.5)	13 (17.1)	93 (20.5)
3 years	40 (17.5)	29 (19.5)	14 (18.4)	83 (18.3)
4 years	39 (17.1)	24 (16.1)	18 (23.7)	81 (17.9)
**Self-reported skin color (n = 453)**				
White	91 (39.9)	83 (55.7)	30 (39.5)	204 (45.0)
Brown	121 (53.1)	62 (41.6)	40 (52.6)	223 (49.2)
Black	13 (5.7)	4 (2.7)	6 (7.9)	23 (5.1)
Yellow	3 (1.3)	–	–	3 (0.7)
**Type of immunization error (n = 55)**				
Inadequate interval between vaccines	24 (54.5)	1 (25.0)	1 (14.3)	26 (47.3)
Vaccine administered outside the recommended age	18 (40.9)	1 (25.0)	4 (57.1)	23 (41.8)
Inadequate interval between doses	2 (4.6)	2 (50.0)	2 (28.6)	6 (10.9)
**Vaccination delay in the booklet (n = 453)**				
Vaccination delayed	183 (80.3)	93 (62.4)	31 (40.8)	307 (67.8)
No vaccination delay	45 (19.7)	56 (37.6)	45 (59.2)	146 (32.2)
**Booklets with vaccination delays and immunization errors (n = 44)**				
Presence of immunization error and vaccination delay	36 (97.3)	3 (75.0)	2 (66.7)	41 (93.2)
Presence of immunization error without vaccination delay	1 (2.7)	1 (25.0)	1 (33.3)	3 (6.8)
**Children’s age at the time of immunization error (n = 55)**				
From 6 months to < 1 year	13 (29.6)	2 (50.0)	5 (71.4)	20 (36.4)
From 1 to 4 years	31 (70.4)	2 (50.0)	2 (28.6)	35 (63.6)

During the period studied, 131,741 doses of vaccines were administered in the age group studied in the three municipalities assessed, and the prevalence of immunization errors found was 41.9 immunization errors for every 100,000 doses applied (95% CI 32.2 – 51.6).

Among the 55 immunization errors found, 5 (9.1%) occurred in 2017, 11 (20.0%) in 2018, 8 (14.5%) in 2019, 16 (29.1%) in 2020 and 15 (27.3%) until October 2021.

The highest prevalence of errors occurred between 2020 (50.0/100,000 doses applied) and 2021 (78.6/100,000 doses applied), according to the number of doses applied in the period.

Table 2 shows the descriptive analysis of errors according to the type of immunobiological agent and dose, with their respective prevalence and type of error. The vaccine administered with the highest prevalence of errors was adsorbed diphtheria, tetanus and pertussis (DTP) vaccine (13.7/100,000), followed by (attenuated) polio vaccine 1,3– oral polio vaccine (OPV) (8.4/100,000). The majority of immunization errors related to DTP and OPV administration occurred during the administration of the 1^st^ booster, carried out at an interval of less than 6 months in relation to the 3^rd^ dose of adsorbed diphtheria, tetanus, pertussis, recombinant hepatitis B and *Haemophilus influenzae* B vaccine (pentavalent) and DTP, inactivated polio vaccine 1, 2, 3 (IPV and OPV).

**Table 2:** Classification of immunization error, vaccines involved, proportion and prevalence, Minas Gerais, Brazil, 2021.

Vaccines involved in immunization error	Immunization error			Proportion of immunization errors (%)	Prevalence (100,000 doses applied) (95%CI*)
	Inadequate interval between doses (n = 07)	Inadequate interval between vaccines (n = 27)	Vaccine administered outside the recommended age (n = 21)		
DTP^†^	–	16	2	32.7	13.7
OPV^‡^	–	11	–	20.0	8.4
CV^§^	–	–	7	12.7	5.3
VRH^||^	1	–	6	12.7	5.3
HepB^¶^	–	–	3	5.5	2.3
IPV**	2	–	1	5.5	2.3
10VPC^††^	2	–	–	3.6	1.5
Pentavalent^‡‡^	1	–	1	3.6	1.5
MMR^§§^	1	–	1	3.6	1.5
Total	7	27	21	100.0	41.9 (32.2 – 51.6)

*CI: Confidence Interval; ^†^DTP: adsorbed diphtheria, tetanus and pertussis vaccine; ^‡^PWV: (attenuated) polio vaccine 1, 3; ^§^CV: chickenpox vaccine; ^||^HRV: G1P1 (attenuated) human rotavirus vaccine [8]; ^¶^HepB: hepatitis B vaccine (recombinant); ^**^VIP: inactivated polio vaccine 1, 2, 3; ^††^10VPC: (conjugate) 10-valent pneumococcal vaccine; ^‡‡^Pentavalent: adsorbed diphtheria, tetanus, pertussis, recombinant hepatitis B and *Haemophilus influenzae* B vaccine; ^§§^MMR: measles, mumps and rubella vaccine (attenuated) – triple viral.

When checking the immunization errors identified on children’s vaccination cards, with reporting in SIEAPV and/or e-SUS *Notifica*, it was observed that all immunization errors identified were underreported by the health services of the participating municipalities.

A statistically significant association was observed between the presence of an immunization error and vaccination delay (Table 3). Children who had delayed vaccination schedules had 7.5 times the chance of suffering an immunization error when compared to children with up-to-date vaccinations.

**Table 3:** Analysis of the association between the presence of immunization errors according to children’s vaccination delay, age, skin color and sex, Minas Gerais, Brazil, 2021.

Variable	Presence of immunization errors n (%)		p-value	Prevalence ratio (95%CI*)
	Yes	No		
**Vaccination delay**				
Yes	42 (13.7)	265 (86.3)	<0.001	7.55 (2.30 – 24.80)
No	3 (2.1)	143 (97.9)		
**Child’s age**				
From 6 months to < 1 year	3 (4.3)	66 (95.7)	0.144	–
1 year	11(8.7)	116 (91.3)		
2 years	15 (16.1)	78 (83.9)		
3 years	9 (10.8)	74 (89.2)		
4 years	7 (8.6)	74 (91.4)		
**Skin color**				
White	21 (10.3)	183 (89.7)	0.504	–
Brown	20 (9.0)	203 (91.0)		
Black	3 (13.0)	20 (87.0)		
Yellow	1 (33.3)	2 (66.7)		
**Sex**				
Female	19 (8.5)	204 (91.5)	0.322	–
Male	26 (11.3)	204 (88.7)		

*CI = Confidence Interval.

The results of this study were presented to the managers of the participating municipalities for discussion and adoption of strategies to minimize the occurrence of immunization errors.

## DISCUSSION

The results of this study demonstrated a high prevalence of immunization errors, with an increase in 2020 and 2021 and an important underreporting of these errors. The most prevalent error was the inadequate interval between vaccines and the highest proportion of errors were related to DTP vaccine administration. Furthermore, vaccination delay was related to the chance of an immunization error occurring. The prevalence of immunization errors in the studied population was higher compared to other studies carried out in Brazil, based on reports in the PNI Information Systems in Goiás (4.05/100,000)^(9)^, Porto Alegre (19.9/100,000)^(22)^, Paraná (19.4/100,000 in the Bacillus Calmette-Guérin vaccine (tuberculosis vaccine) – BCG)^(1)^ and Goiânia/GO (0.6/10,000 in DTP vaccine)^(2)^. The findings of this study reaffirm the underreporting of immunization errors in health services.

Underreporting is a public health problem that prevents real knowledge of occurrence of errors in the vaccination process^(1)^, in addition to favoring the resurgence of vaccine-preventable diseases in the community, when revaccination is not carried out in cases where it is necessary(11).

Investigations carried out in Brazil, South Korea and the United States^(9–11,23)^ also pointed to underreporting of immunization errors, making it difficult to adopt preventive measures. A systematic review study, assessing the prevalence of immunization errors worldwide, identified that studies that used active surveillance systems or processes observed a higher prevalence of immunization errors when compared to studies that used spontaneous reports^(24)^.

An integrative literature review that aimed to analyze the reasons for not reporting patient safety incidents identified underreporting of errors in 87.5% of studies assessed. One of the main reasons for underreporting is related to punitive culture and health professionals’ fear of being penalized or reprimanded^(25)^. This punitive culture can lead to omission of reporting events/errors and make it difficult to build an institutional patient safety culture. The fear and embarrassment of reporting errors should be considered a point of attention and improvement in health services(14).

Furthermore, the slowness of information systems for reporting, work overload, lack of knowledge of the importance of filling out the immunization error investigation form and the lack of adequate training to fill it out correctly may be contributing factors to underreporting^(10,12,25)^, even for those errors without ESAVI.

The need for completeness of reporting data is highlighted in order to develop prevention and control strategies and, consequently, improve quality of care^(14)^. Professionals must be made to feel safe and valued to understand the importance of reporting in providing opportunities and contributing to strengthening patient safety in health services^(25)^. The most prevalent errors were inadequate interval between vaccines, followed by vaccines administered outside the recommended age. Studies also corroborated these findings^(9,11,18,23)^. Health professionals’ lack of knowledge about the recommended intervals and minimum intervals between vaccines exposes them to immunization errors. And this, when combined with underreporting, contributes to the maintenance and perpetuation of these events in vaccination rooms(1).

The appropriate vaccination schedule, with recommended ages, number of doses and intervals between doses, is based on clinical studies and the characteristics of each immunizing agent^(6,20,26)^. Such intervals, described in the vaccination calendar, particular to each vaccine, are necessary and essential for reducing antibodies produced by the previous dose, ensuring the effectiveness of vaccination after completion of the vaccination schedule^(6,20,26)^.

An investigation conducted in the United States pointed out that the complexity of the vaccination schedule, the diversity of vaccines and the similarity between them may have contributed to administration of vaccines outside the recommended age, more frequently in infants and children^(3)^. Furthermore, a study carried out in France identified a partial domain of knowledge about vaccines among the health professionals interviewed, with persistent gaps that can significantly contribute to occurrence of errors in the vaccination process(8).

The vaccine with the highest incidence of immunization errors was DTP. It is possible to assume that the increase in errors with this vaccine is related to vaccination delay caused by the shortage of Pentavalent vaccine in Brazil in 2019^(27)^. The minimum interval between the 3^rd^ dose of Pentavalent and the first DTP booster is 6 months. If health professionals are not well informed about the intervals between doses and do not observe the minimum recommended time for administering the vaccine, this could result in immunization errors.

The highest immunization error rates occurred in 2020 and 2021 and vaccination delays were associated with an increase in errors. The relationship between vaccination delays and the eventuality of immunization errors becomes worrying, as the COVID-19 pandemic severely influenced the vaccination routine, especially during 2020.

Research carried out to assess the impact of the pandemic on vaccination coverage rates in children under one year old in Brazil identified that from 2019 to 2020 vaccination coverage reduction was 11.1% on average, much higher than previous variations that were around six percentage points. Furthermore, the individual analysis per vaccine showed an even higher value, as in the case of HepB, around 20.4%^(28)^. Considering the impact on vaccination coverage, it is likely that many delayed cards will reach health services and, if professionals do not follow the recommended standards and intervals, the chance of an immunization error occurring may be increased.

The need for strategies to prevent immunization errors is clear. International study points to the use of a vaccine administration checklist, which facilitates application and provides safe preparation/administration^(29)^. Another alternative is investments in information technology infrastructure, identifying risk factors for immunization errors, and continuing education based on daily routine in vaccination rooms and on mistakes already made, generating reflections and learning for professionals. Furthermore, it is necessary to involve individuals in the vaccination procedure so that they serve as a barrier to error, detecting process failures when identifying individuals and checking the vaccine administered^(9)^. Associated with this, it is essential to improve and invest in strategies that ensure that vaccines are always available, in order to avoid delays in administration and prevent possible immunization errors.

In vaccination rooms in public health services in Brazil, nursing takes responsibility and can be considered the last barrier to intercepting an immunization error. It is impossible to completely eliminate the risks of immunization errors, however identifying their causes is essential for acquiring new knowledge that makes it possible to propose interventions, aiming at safety in the vaccination room.

In this regard, the importance of a nursing team with up-to-date knowledge about the vaccination calendar, recommended vaccination schedule for each age, number of doses and intervals between doses is reinforced, in addition to the constant supervision of nurses in carrying out routines in the vaccination room to change this scenario. Nurses’ distance from the vaccination room contributes to precarious training and updating, as supervision allows identifying the vaccinator’s difficulties and, consequently, continuing education^(4)^. Therefore, the supervision and technical responsibility of nurses in vaccination activities^(14)^ are essential to guarantee the quality of care provided.

The methodological quality of this study enabled a direct search for data, based on children’s vaccination records, allowing greater knowledge of immunization errors and their underreporting. As a limitation of this study, the study design used must be considered, since cross-sectional studies represent observations and outcomes at a single moment in time. Therefore, from the vaccine cards, it was not possible to identify errors related to vaccine administration and handling. This fact may underestimate the prevalence of immunization errors. However, the strength of this study is the novelty of investigating immunization errors using primary data and comparing them with the database of reports of immunization errors in the state, confirming the presence of underreporting.

## CONCLUSION

The results of this study point to a high prevalence of immunization errors found in children’s vaccination records and their underreporting. This investigation therefore encourages discussion on the need to reinforce the importance of adopting preventive measures against immunization errors, maintaining the guarantee of safe vaccination for users and leading to the success of immunization actions.

The results found also reaffirm that underreporting of immunization errors is a reality to be faced in health services, and, for a better understanding of the factors associated with underreporting of these events, it is necessary to carry out more in-depth studies on the subject.

## Data Availability

The data is available in the Mendeley Data repository under DOI: https://www.doi.org/10.17632/b57ytpv4ws.1

## References

[1] Bisetto LHL, Ciosak SI (2017). Analysis of adverse events following immunization caused by
immunization errors.. Rev Bras Enferm.

[2] Braga PCV, Silva AEBC, Mochizuki LB, Lima JC, Sousa MRG, Bezerra ALQ (2017). Incidence of post-vaccination adverse events in
children.. Rev Enferm UFPE On-Line..

[3] Rodgers L, Shaw L, Strikas R, Hibbs B, Wolicki JE, Cardemil CV (2018). Frequency and cost of vaccinations administered outside minimum
and maximum recommended ages: 2014 data from 6 sentinel sites of
immunization information systems.. J Pediatr.

[4] Oliveira VC, Tavares LOM, Maforte NTP, Silva LNLR, Rennó HMS, Amaral GG (2019). A percepção da equipe de enfermagem sobre a segurança do paciente
em sala de vacinação.. Rev Cuid (Bucaramanga).

[5] Samad F, Burton SJ, Kwan D, Porter N, Smetzer J, Cohen MR (2021). Strategies to reduce errors associated with 2-component
vaccines.. Pharmaceut Med.

[6] Brasil. Ministério da Saúde. (2021). Manual de vigilância epidemiológica de eventos adversos pós-vacinação
[Internet]..

[7] Wolicki J, Miller E, Vaccine administration. Centers for Disease Control and
Prevention (2021). Epidemiology of Vaccine Preventable Diseases [Internet]..

[8] Poiraud C, Réthoré L, Bourdon O, Lorrot M, Prot-Labarthe S (2023). Understanding and preventing vaccination errors.. Infect Dis Now..

[9] Barboza TC, Guimarães RA, Gimenes FRE, Silva AEBC (2020). Retrospective study of immunization errors reported in an online
information system. Rev Latino-Am Enfermagem.. Rev Lat Am Enfermagem.

[10] Pacheco FC, Domingues C, Maranhão A, Carvalho S, Teixeira A, Braz R (2018). Análise do sistema de informação da vigilância de eventos
adversos pós-vacinação no Brasil, 2014 a 2016.. Rev Panam Salud Publica.

[11] Suragh TA, Hibbs B, Marquez P, McNeil MM (2020). Age inappropriate influenza vaccination in infants less than 6
months old, 2010–2018.. Vaccine.

[12] Santos LCB, Silva HS, Borja-Oliveira CR, Chubaci RYS, Gutierrez BAO (2021). Eventos adversos pós-vacinação em idosos no Estado de São Paulo,
Brasil, de 2015 a 2017.. Cad Saude Publica.

[13] Silva TPR, Silva SF, Dutra MM, Silva RB, Gusmão JD, Matozinhos FP (2021). Analysis of immunization errors in pregnant
women.. Rev Esc Enferm USP.

[14] Françolin L, Gabriel CS, Bernardes A, Silva AEBC, Brito MFP, Machado JP (2015). Patient safety management from the perspective of
nurses.. Rev Esc Enferm USP.

[15] Couto MT, Barbieri CLA, Matos CCSA (2021). Considerations on covid-19 impact on the individual-society
relationship: from vaccine hesitancy to the clamor for a
vaccine.. Saude Soc.

[16] Minas Gerais. Secretaria de Estado de Saúde de Minas
Gerais. (2019). Deliberação CIB-SUS/MG N^o^ 3.013, de 23 de outubro de 2019.
Aprova o Ajuste/2019 do Plano Diretor de Regionalização PDR/SUS-MG e dá
outras providências [Internet]..

[17] Instituto Brasileiro de Geografia e Estatística. (2021). População por cidades [Internet].

[18] Donnini DA, Silva CMB, Gusmão JD, Matozinhos FP, Silva RB, Amaral GG (2022). Incidence of immunization errors in the state of Minas Gerais,
Brazil: a cross-sectional study, 2015–2019.. Epidemiol Serv Saude.

[19] Ferreira VLDR, Waldman EA, Rodrigues LC, Martineli E, Costa ÂA, Inenami M (2018). Avaliação de coberturas vacinais de crianças em uma cidade de
médio porte (Brasil) utilizando registro informatizado de
imunização.. Cad Saude Publica.

[20] Brasil. Ministério da Saúde. (2020). Instrução normativa referente ao calendário nacional de vacinação 2020
[Internet].

[21] Brasil. Ministério da Saúde. (2019). Manual dos Centros de Referência para Imunobiológicos Especiais
[Internet]..

[22] Capponi RL, Cunha CBS, Paz NS (2020). Avaliação das notificações de erros programáticos na
administração de imunobiológicos em Porto Alegre - RS, 2019.. Rev Eletrônica Acervo Saúde..

[23] Hwa Lee Y, Harris RC, Won Oh H, Oh Y, Vargas-Zambrano JC, Choe YJ (2021). Vaccine-related errors in reconstitution in South Korea: a
national physicians’ and nurses’ survey.. Vaccines (Basel).

[24] Morse-Brady J, Marie Hart A (2020). Prevalence and types of vaccination errors from 2009 to 2018: a
systematic review of the medical literature.. Vaccine.

[25] Alves MFT, Carvalho DS, Albuquerque GSC (2019). Barriers to patient safety incident reporting by Brazilian health
professionals: an integrative review.. Cien Saude Colet.

[26] Luman ET, Barker LE, Shaw KM, McCauley MM, Buehler JW, Pickering LK (2005). Timeliness of childhood vaccinations in the United States: days
undervaccinated and number of vaccines delayed.. JAMA.

[27] Brasil. Ministério da Saúde. (2019). Nota Informativa n^o^ 190/2019 - CGPNI/DEIDT/SVS/MS..

[28] Procianoy GS, Rossini F, Lied AF, Jung LFPP, Souza MCSC (2022). Impact of the COVID-19 pandemic on the vaccination of children 12
months of age and under: an ecological study. Cienc Saude Coletiva.

[29] Charles R, Vallée J, Tissot C, Lucht F, Botelho-Nevers E (2016). Vaccination errors in general practice: creation of a preventive
checklist based on a multimodal analysis of declared errors.. Fam Pract.

